# Nation-wide assessment of the distribution and population size of the data-deficient nurse shark (*Ginglymostoma cirratum*)

**DOI:** 10.1371/journal.pone.0256532

**Published:** 2021-08-24

**Authors:** Francesco Garzon, Rachel T. Graham, Ivy Baremore, Dan Castellanos, Hilmar Salazar, Cynthia Xiu, Zeddy Seymour, Matthew J. Witt, Lucy A. Hawkes

**Affiliations:** 1 MarAlliance, Sal Rei, Ilha da Boavista, Cabo Verde; 2 MarAlliance, Ancon, Panama City, Panama; 3 MarAlliance, Belize City, Belize; 4 Hatherley Laboratories, College of Life and Environmental Sciences, University of Exeter, Exeter, United Kingdom; Institut de Recherche pour le Developpement, FRANCE

## Abstract

The study presents the first national assessment of a nurse shark *(Ginglymostoma cirratum*) population, conducted using a combination of transect surveys and baited remote underwater videos (BRUVs). Density of nurse sharks in Belize was found to be higher in reefs than in lagoons, and in the atolls furthest away from the mainland and human settlements. Only large and old protected areas were found to have a positive impact on nurse shark abundance. Absolute abundance of nurse sharks was estimated using distance sampling analysis, giving a total nurse shark population in the range of 3,858 to 14,375 sharks. Thanks to a vast area of suitable habitat for nurse sharks in the country and legislation already in place for the safeguard of the species, Belize could represent an important hotspot for nurse sharks in the Western Atlantic. The data presented here hence offers a baseline for the long-term monitoring of the Belizean nurse shark population and improves our understanding of nurse shark abundance and distribution in the wider Caribbean basin.

## Introduction

Elasmobranch populations worldwide have suffered considerable declines in the past decades [1], leading to the loss of more than 50% of individuals in many species [2,3]. As a result, this taxon has become the second most threatened among vertebrates, after amphibians [4]. Despite this, many shark species remain poorly studied: 47% of the elasmobranch species in the IUCN Red list of endangered species are classified as data deficient [4], and population trends of 77% of listed shark species remain unknown (IUCN, 2018). The vast geographic extent of marine ecosystems and the highly mobile nature of most marine animals often hinder the ability of researchers to adequately monitor their populations, thus reliable estimates of population size are difficult to obtain for many marine species. Hypotheses and predictions of population declines can nonetheless be put forward on the basis of observed ongoing threats to the species. For fish species, fishing effort, documented landings, and size distribution of caught individuals have often been used to infer population trends [5–9]. However, data on shark catches (legal, illegal, or unreported) is often missing or unreliable [10,11], therefore inferring population trends from such indicators is likely to yield imprecise predictions. Obtaining accurate estimates of abundance from fishery-independent surveys therefore remains critical for a reliable assessment and management of elasmobranch populations [11,12].

The nurse shark (*Ginglymostoma cirratum*) is a widespread elasmobranch species that can be found throughout the tropical and subtropical Atlantic and Eastern Pacific Ocean, ranging from Gabon to the Cape Verde Islands in the West Africa, southern Brazil to North Carolina in the Western Atlantic, and Peru to southern Baja California in the Pacific [13,14]. Despite being widespread [14] and reported as one of the most abundant shark species in coastal shallow waters [14–18], little is known about the ecology and population status of this species [14]. The nurse shark is listed globally as a ‘data deficient’ species by the IUCN [13], and the western Atlantic subpopulation is considered ‘near threatened’ [19] due to ongoing fishing pressure and habitat degradation, which are thought to have caused significant declines in the population throughout the region [19]. Directed fisheries for nurse sharks are known to exist in Panama [19] and Colombia [19], and there is also knowledge of nurse sharks being captured in other parts of the Caribbean for their skin or for the ornamental fish trade [19]. In contrast, fishing of nurse sharks is illegal in Brazil (Annex I of Normative Instruction #05 from the Ministry of the Environment, 2004) and Belize (Government of Belize, 2011); The U.S.A. have introduced size and catch limits for this species; and The Bahamas, Cayman Islands, Bonaire, Saba, Saint Maarten, British Virgin Islands, Dominican Republic, and Honduras have all declared themselves ‘shark sanctuaries’ and prohibited all commercial shark fisheries. Other countries, such as Colombia, are also considering a ban on nurse shark fishing, as well as enacting an extensive habitat protection campaign [19]. Nonetheless, nurse sharks continue to be captured in both Honduras and Belize (R. Graham pers. obs) and incidental and illegal catching of nurse sharks probably persists across other countries in the species’ range [20].

Nurse sharks are also threatened indirectly by habitat degradation and destruction of coastal ecosystems including sandy flats, seagrasses and mangroves [13], and especially on reef systems which constitute their main habitat [21,22]. Indeed, coral reefs in the Western Atlantic are thought to be the most degraded on the planet [23] and losses of up to 80% of coral cover have been documented in the Caribbean [24].

There have been limited studies on the biology of nurse sharks [25–27]. To date, these have suggested that nurse sharks have a long lifespan [22], late maturity [25], and a biennial reproductive cycle [22], meaning that even moderate increases in mortality deriving from fishing activities or habitat degradation could cause rapid population declines [28]. Long-distance movements (>300km) and migratory patterns have been observed in some nurse shark populations [29,30]. In most cases, however, nurse sharks appear to display strong site fidelity [22,31] and breeding site philopatry [26], with many tracked individuals only moving within less than 10km from where they were first sighted after months at liberty [29,32]. In Belize, high residency levels were encountered among nurse sharks acoustically tracked in and around Glover’s Reef Marine Reserve, where the mean dispersal distance for this species was 7.7km (although movements >10km were sporadically encountered too [32]). Due to their resident nature, the disappearance of nurse sharks from certain areas may result in permanent regional extinctions [33]. In order to avoid further local extinctions of nurse sharks in other regions, it is important to monitor the population trend of this species. At present, no regional or national estimates of population size are available for nurse sharks in any of the countries encompassed by this species’ range except for localized site estimates for Atol das Rocas, Brazil (about 6 km^2^ in area), where Mark-Recapture models yielded estimates of 339 to 368 individuals [16]. This lack of baseline information hinders efforts to compile a thorough assessment of nurse shark populations, while direct and indirect threats persist throughout their range. More information on the abundance and distribution of the species is urgently needed to inform appropriate conservation plans.

Belize hosts the world’s second longest (~250 km) barrier reef, and one of the healthiest reef ecosystems in the western Atlantic [23]. Belize could therefore be an important hotspot for nurse shark conservation. Nurse sharks in Belize are also an important touristic attraction, especially in the northern Cayes where three ‘provisioning sites’ (where sharks are fed by tour companies for visitors to snorkel with them) generate considerable income for local businesses (RTG, unpublished data). Although the importance of nurse sharks to the Belizean tourist industry and the threat that fishing poses to this species have been recognised, very little information is available concerning the abundance, distribution, and conservation status of the species in Belize despite the legislated ban on fishing nurse sharks (Government Of Belize, 2011). Accurate knowledge on the abundance and distribution of nurse sharks in the country is necessary to monitor the population trend of the species and evaluate the efficacy of the present regulation at providing protection for this species.

Here, we report the first country-wide estimate of nurse shark abundance and distribution in Belize, indeed the first for any nation throughout the species’ range. Baited remote underwater videos (BRUVs) and in-water visual census transects were used to survey multiple locations of the Belizean barrier reef and all three offshore atolls to census the local nurse shark population. The aims of the study were to 1) obtain a first, approximate estimate of the number of nurse sharks in Belize; and 2) describe patterns and drivers of their distribution and abundance along the Belizean barrier reef.

## Methods

This work was approved by the relevant local authorities (the Belize Fisheries Department).

### Study regions and locations

**Fig 1:**
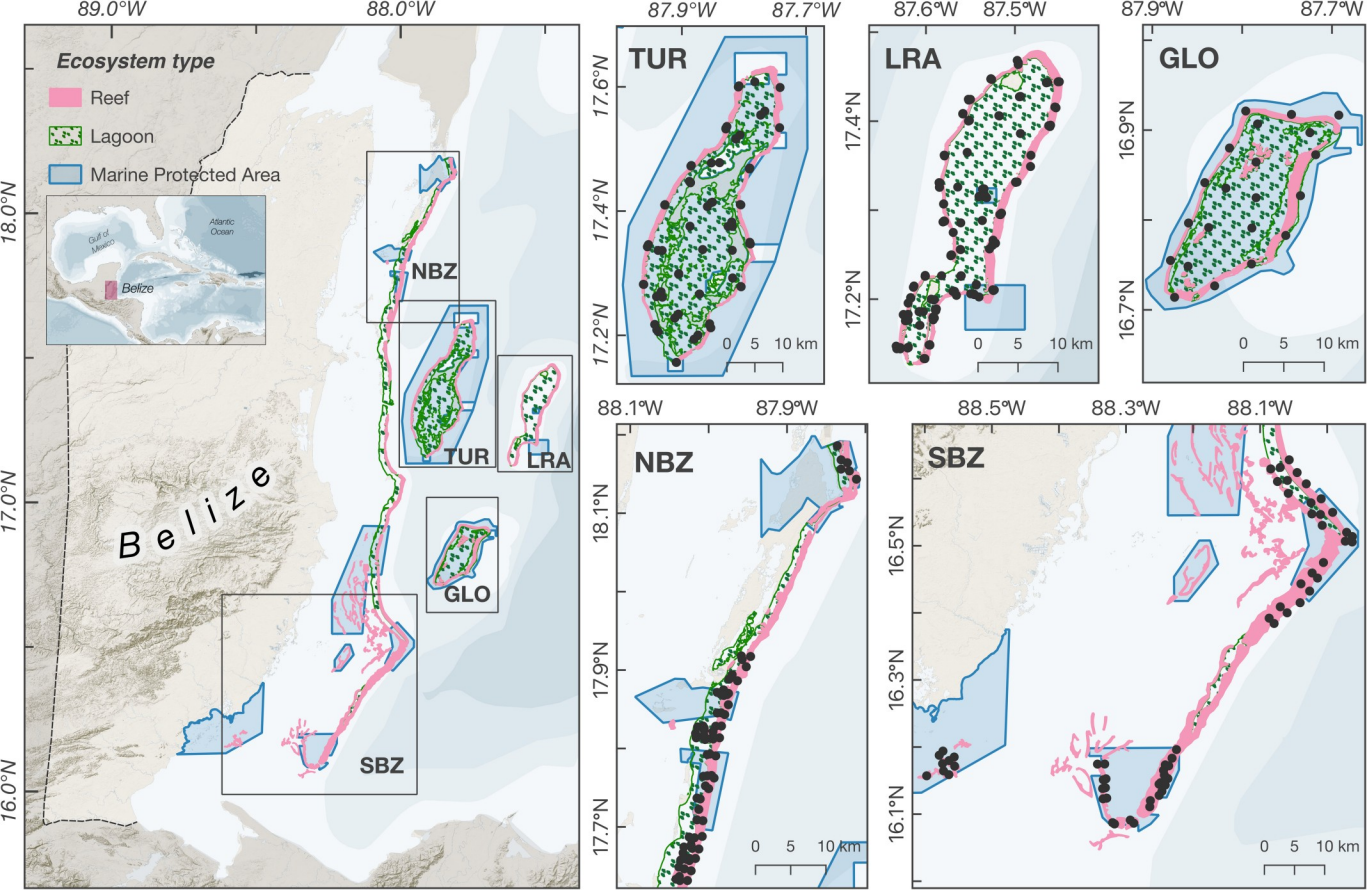
Survey set-up for the assessment of nurse shark (Ginglymostoma cirratum) distribution and abundance in Belize. Study regions covered by BRUVs and transect surveys: Northern Belize (NBZ), Turneffe Atoll (TUR), Lighthouse Reef (LRA), Glovers Reef (GLO) and Southern Belize (SBZ). Black dots represent survey locations (deployment site for BRUVs and starting points for transects). Base layer created in MapBox Studio (https://studio.mapbox.com) using freely available data from MapBox (hillshade and terrain data; can be found within the software) and Natural Earth (www.naturalearthdata.com; bathymetry and geopolitical contours). Ecosystem shapefile data was obtained from http://www.biodiversity.bz (2015 version; [36]) and MPA boundaries were constructed using the legal descriptions of the boundaries as published on the Government Gazette Statutory Instruments.

Five study regions, covering key parts of the Belizean reef that were anecdotally known to host nurse sharks, were surveyed: Northern Belize (NBZ), Southern Belize (SBZ), Turneffe Atoll (TUR), Lighthouse Reef Atoll (LRA), and Glover’s Reef (GLO; Fig 1). Within each region, survey locations were determined using a stratified random methodology that was designed to cover all the main macrohabitats found in the regions (forereef, backreef, patch reef, and seagrass beds; Fig 1) and differentiate between protected and unprotected areas. Surveys were conducted between 2012 and 2017, and whenever a region was surveyed in more than one year, the same locations were visited. Survey locations were placed both inside and outside marine protected areas (117 and 134 locations, respectively). The effectiveness of protected areas is largely dependent on their size, age, and level of enforcement [34], and it has been suggested that particularly large protected areas (containing >50 km of reef) could be required for the effective conservation of nurse sharks [35]. To account for these factors in the analysis of the effects of spatial protection in nurse shark abundance, four categories were created to describe the MPAs present along the Belizean barrier reef, based on their size and age: old (>20 years old) and large (>20 km of reef; Glover’s reef Marine Reserve and Sapodilla Caye MR), old and small (<20km of reef; Blue Hole and Half Moon Caye National Monuments, Hol Chan MR, and Bacalar Chico MR), new (<20 years old) and large (Turneffe Atoll MR and Port Honduras MR), and new and small (Caye Caulker MR, Southwater Caye MR, Gladden Spit MR).

As proximity to human population centres has been associated with negative impacts on wildlife abundance and ecosystem quality [37,38], the “human gravity” associated with each study location was obtained from Cinner et al. (2018) [38]. Human gravity was created as a concept to quantify the interaction between human and coral reef fish [38], and provides a proxy measurement of the likely human impact on any given coral reef, based on the distance and size of human settlements. The mean (average) human impact gravity of each region was also calculated as the mean of the impact gravity scores extracted at each survey site within a region.

### Data collection

#### Region area estimation

To calculate the area of each region, the extent of likely suitable habitat within them was determined. As nurse sharks do not generally occupy open water [22,39], only the extent of Reef ecosystem (comprising forereef and backreef), and the Lagoon ecosystem (patchreef and seagrass beds) were used for area estimation. The geographic extent of each ecosystem was enumerated from a spatially referenced map of the ecosystems of Belize (2015 version; [39]). Previous BRUV surveys conducted in the country suggest that nurse sharks are absent from lagoon areas further away from reef systems (FG unpublished data). For NBZ and SBZ, the Lagoon ecosystem extended to areas not covered by the surveys but unlikely to host nurse sharks; for these reasons, the Lagoon ecosystem in these areas was restricted to a strip delimited by the reef edge and stretching to a maximum of 2 km landward, although we acknowledge that this arbitrary threshold may be imperfect.

#### Baited remote underwater videos

Nurse shark relative abundance was surveyed using Baited Remote Underwater Videos (BRUVs). BRUVs have been previously found to produce comparable estimates of abundance to those obtained from underwater visual census and baited hook and line methods [40,41], and to be well suited to sampling generalist and large carnivores [42]. BRUVs consisted of a GoPro camera mounted on a metal frame, with a wire mesh bag of crushed baitfish suspended 110cm in front of the GoPro. A 30cm long metal bar was fixed perpendicularly above the bait, to allow length estimations of nurse sharks to be made (see [41] for more information on BRUVs). BRUVs were deployed from a boat using a rope and in-water operators guided it away from live coral. Each BRUV was left to record continuously for at least 65 minutes after settling on the sea floor and manually retrieved afterwards using a small marker float attached to the rope used to lower the BRUV into position. Time of deployment, GPS location, bottom depth, type of bait used, habitat type (forereef, backreef, patchreef, or seagrass beds), and positioning within or outside MPA borders were recorded for each BRUV deployment. BRUVs were deployed at study regions from 2012 to 2016 (Table 1). A total of 409 successful BRUV deployments were made during the course of the surveys across all regions. BRUV deployments were equally distributed within and outside protected areas (204 and 205 deployments, respectively); 245 BRUVS were deployed in reef ecosystems and 164 in lagoon ecosystems.

**Table 1:** Summary statistics of surveys.

Survey type	Statistic	GLO	LRA	NBZ	SBZ	TUR
	MPAs	Glover’s reef MR	Blue Hole NM, Half Moon Caye NM	Hol Chan MR, Caye Caulker MR, Bacalar Chico MR	Southwater Caye MR, Sapodilla Caye MR, Port Honduras MR	Turneffe Atoll MR
	Mean Human Impact Gravity (standard error)	76.19 (1.31)	50.92 (0.40)	61.81 (1.27)	69.53 (2.20)	126.94 (2.02)
Area estimation (km^2^)	Reef Area	45.36	47.65	30.70	235.46	68.67
	Lagoon Area	201.63	222.52	50.58	40.87	425.702
	Total Area	246.99	270.17	81.28	276.33	494.372
BRUV	Survey years	2016	2012, 2014–2016	2012	2012	2014–2016
	Tot BRUVs	21	170	74	63	81
	Positive BRUVs	10	25	13	10	6
	Nurse sharks recorded	15	32	15	11	6
	Mean MaxN (standard error)	0.476 (0.112)	0.132 (0.028)	0.203 (0.054)	0.175 (0.053)	0.091 (0.039)
	FO	0.476	0.357	0.176	0.159	0.193
Transects	Survey years	/	2012, 2014–2017	2012	2012	2014–2016
	Tot transects	/	223	53	59	78
	Positive transects	/	86	5	15	9
	Nurse sharks recorded	/	162	5	19	14
	Mean ER (standard error)	/	12.96 (1.73)	1.57 (0.68)	5.37 (1.36)	3.10 (1.28)

Summary statistics of nurse shark (*Ginglymostoma cirratum*) surveys, including: Area estimation, BRUV (Baited Remote Underwater Video) deployments, and transect surveys for all study regions (GLO = Glovers Reef; LRA = Lighthouse Reef Atoll; NBZ = Northern Belize; SBZ = Southern Belize; TUR = Turneffe Atoll). MPA = Marine Protected Areas; Positive refers to the count of BRUVs or transects in which a nurse shark was sighted; FO = Frequency of Occurrence, expressed as the number of positive BRUVs over the number of BRUVs deployed; ER = Encounter Rate, expressed as sharks per km^2^.

#### Snorkel transects

Snorkel transects were conducted at NBZ and SBZ in 2012, LRA in 2012 and 2014–2017, and at TUR in 2012 and 2014–2016 (Table 1). No transects were conducted at GLO. Snorkel transects involved four snorkelling observers (trained in species identification and distance/size estimation prior to the surveys) in the water and at least one observer directing swimmers from a boat. Snorkel observers were lined up at a distance of 15m from each other, and each surveyed a parallel 15m wide section of water (7.5m on either side of the observer, totalling 60 metres wide for all four observers) for nurse sharks at similar speed, in a designated direction, for 1 km. Boat-based observers ensured that the transect were swum in a straight line and that inter-observer distances were maintained throughout the survey. If visibility dropped below 15m, transect width was reduced and accounted for in calculations. For each nurse shark encountered, snorkel observers recorded the perpendicular distance from the transect line and the approximate size of the nurse shark. To avoid duplicate sightings, snorkel observers signalled observations to the person on either side of them. Boat-based observers recorded GPS positions of the beginning, middle, and end points of the transect, as well as the distance covered by the snorkel observers.

### Data analysis

#### MaxN and frequency of occurrence

The videos obtained from BRUVs were analysed independently by two observers to minimise identification errors. Observers discarded the first 5 minutes of each video following the BRUV landing on the seafloor to allow for it to settle and boat disturbance to be minimized, and then watched the subsequent 60 minutes of each video, recording the time of arrival of any nurse sharks (i.e. time elapsed from BRUV deployment) and, when possible, an estimate of the length and sex of each nurse shark that appeared in the frame. The maximum number of individuals observed in a single frame (MaxN) in each video was also noted. The average MaxN and frequency of occurrence (FO; i.e. the proportion of BRUVs in which nurse sharks were sighted over the total number of BRUVs deployed) of BRUV deployments were generated for each of the five major study regions, sites within and outside protected areas, and sites within reefs and lagoons. As the vast majority (99%) of BRUVs had a MaxN of either 0 or 1 (range 0–4), the presence or absence of nurse sharks from each BRUV was recorded, and this metric was used in subsequent analyses instead of MaxN. For those regions that were surveyed in multiple years (LRA and TUR), multiple BRUV drops in the same location were grouped to form a unique observation with MaxN equal to the average MaxN recorded in all drops, and a presence/absence (PA) value which was assigned a “1” (i.e. nurse shark present) if nurse sharks were sighted at the location in any of the BRUVs deployments across survey years. The effect of region, protected area category, ecosystem type, depth, and human impact gravity on the likelihood of nurse sharks being sighted in BRUVs were investigated using generalised mixed models (GLMMs) with binomial family distribution and logit link function. All possible combinations of explanatory variables were tested, and the best fit model was selected as that having the lowest AIC score. When two models had similar AIC scores (ΔAIC<2), the most parsimonious model (i.e. the model with the lowest number of explanatory variables) was selected (S1 Table). Post-hoc analyses were conducted using Tukey test with Holm correction for multiple comparisons.

#### Encounter rate and distance sampling analysis

The number of nurse sharks recorded in each transect was used to calculate encounter rate (ER, sharks km^-2^), expressed as number of sharks (n) divided by the area covered by the transect (number of transects x number of observers per transect x length of transect x width of transect). For stations visited in multiple years, ER was computed as the mean of all transects conducted at that location. Mean ER was calculated for each study region, ecosystem type, and protected versus non protected areas and used as an index of nurse shark density. Differences in ER between ecosystems and protected and non-protected areas were then compared using Wilcoxon-Mann-Whitney U test. Regional variation in ER was investigated using a Kruskall-Wallis test. The Dunn test with Holm correction for multiple comparisons was used to conduct post-hoc multiple comparisons of ER between regions. As the probability of sighting an animal logically decreases as the distance from the observer increase, distance sampling analysis was employed to estimate density while correcting for the chance that nurse sharks were present but not recorded. Distance sampling analysis is a widely used methodology of density estimation, which is centred on detection functions [43,44]. Detection functions describe the probability of an animal being seen as a function of its distance from the transect line, and is used to correct density estimates to account for unrecorded individuals.

**Fig 2:**
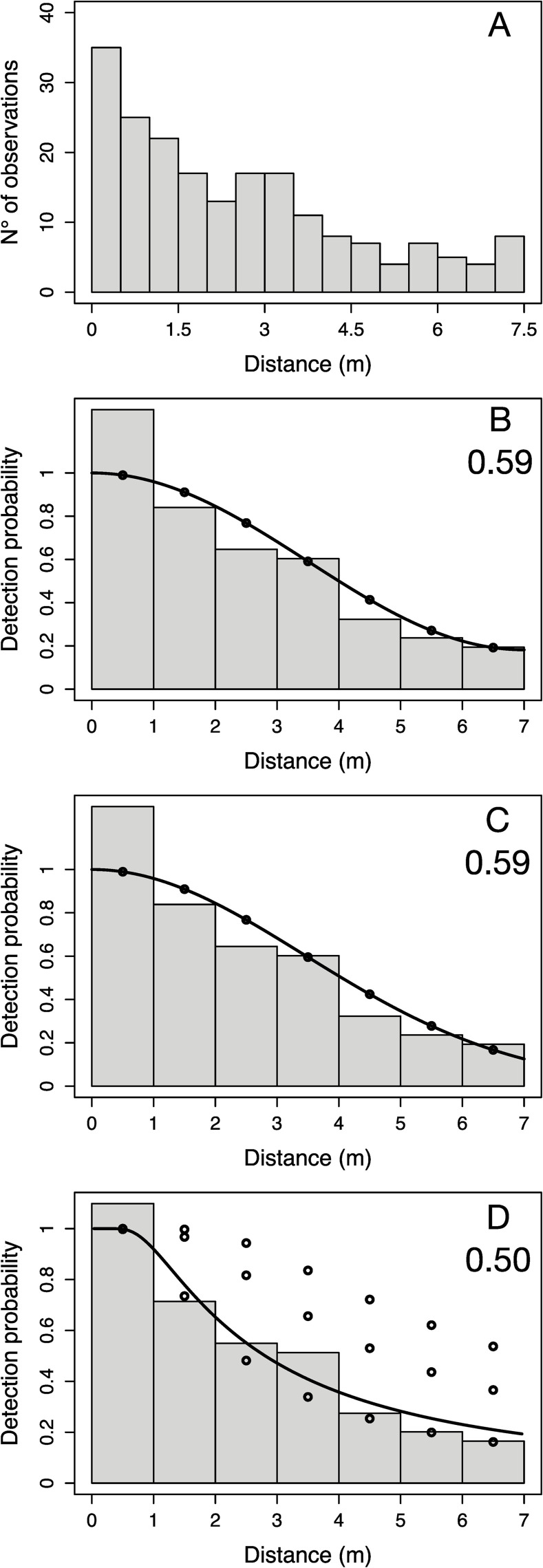
Key functions used for the estimation of abundance of nurse sharks (*Ginglymostoma cirratum*) from transect data. (A) Histogram of recorded perpendicular distances in transect surveys (before transformation for distance sampling analysis), key functions fitted in distance sampling analysis (B) uniform function with cosine adjustment, (C) half-normal function, and (D) hazard-rate function with ecosystem type as covariate. Detection probabilities associated with each function are presented in the top-right corner.

In this study, sightings from all survey areas, years, sexes, and habitats were pooled together in order to have enough data to reliably estimate the probability density function. Distance sampling is robust to pooling data, and the practice likely does not lead to biased results [45]. Consequently, the number of sharks recorded was plotted against distance from the observer and inspected to assess the assumption that distances were accurately recorded (i.e. not rounded to the closest convenient number). A tendency for rounding of distances to the nearest unit (e.g. 0, 1, 3 metres) could be detected (Fig 2). Hence, to improve fit of the detection function, data were pooled into seven equal-sized bins with right truncation (distance<7m). After truncation, 192 nurse shark sightings were available for the estimation of the probability density function. Optimal model fits were investigated [46] and a variety of key functions and adjustment term combinations (uniform + cosine or simple polynomial, half-normal + cosine or simple polynomial, hazard rate + cosine or hermite polynomial) were fitted. Akaike’s Information Criteria (AIC) and chi-square goodness of fit tests were used for model selection, with particular attention paid to model fit at distances near zero since the fit of the shoulder near zero is most important for robust estimation [43]. The Distance package [47] for the statistical software R was used. Three functions (uniform with cosine adjustment term of 1^st^ order, half-normal with no adjustment term, and hazard rate function with ecosystem type fitted as a covariate) had similar goodness of fit and AIC scores, and were therefore all selected to conduct the analysis (Fig 2). Following this process, nurse shark density (D) within each study area was then estimated following [43] as:

D=nf(0)/2L

where n corresponds to the total number of sharks sighted, f(0) is the probability density function of the perpendicular distances evaluated at zero distance, and L represents the total transect effort (number of transects x number of observers per transect x length of transect). The analysis was re-run for reefs and lagoons within each region estimating abundance by multiplying D by the area of each ecosystem found in the region of interest.

#### National population size estimation

A conservative estimate of total nurse shark abundance was estimated by assuming that all nurse sharks that were present in the area covered by transect surveys were recorded. Under this assumption, the average encounter rates per unit area recorded across all transects in reefs and lagoons were calculated and multiplied by the total area of each ecosystem in Belize. This number should represent a minimum estimate of nurse sharks in Belize as it is scaled from the number directly observed, whereas in reality many more may have been in obscure habitats or too deep to observe.

Some nurse sharks were likely not seen during transects because the distribution of sightings was not uniform against distance from the observer (Fig 2A). Thus, an upper estimate of the number of nurse sharks in Belize was also generated by using the corrected densities obtained from the distance sampling analysis. In order to obtain population size estimates for the regions of Belize that were not covered by transect surveys, the density estimates obtained for reef and lagoon ecosystems in each survey region were averaged separately for each of the three detection functions. The total population size was then estimated by multiplying each density estimate obtained from the three detection functions by the total area of reef and lagoon ecosystem present in the region. For each region, the highest estimates of density in reef and lagoons were used in order to obtain a maximum estimate of abundance.

## Results

### Estimation of the area of the study regions

The total amount of reef and lagoon habitat assumed to likely host nurse sharks in Belize was estimated to be 1,811 km^2^. The five regions covered by this study encompassed 75% (1,369km^2^) of the total habitat available, including 428 km^2^ of reef ecosystem and 941 km^2^ of lagoon ecosystem (Table 1).

The average human impact gravity of the regions differed significantly (Kruskall-Wallis test, χ^2^_4_ = 369.76, p<0.01), with SBZ having a significantly and considerably higher mean impact gravity than all other regions (Dunn test, p<0.01; Table 1). LRA had the lowest average impact gravity than all regions (50.92; Dunn test, p<0.01), while NBZ, TUR, and GLO had similar and intermediate impact gravity scores (Table 1).

### MaxN and FO

**Fig 3:**
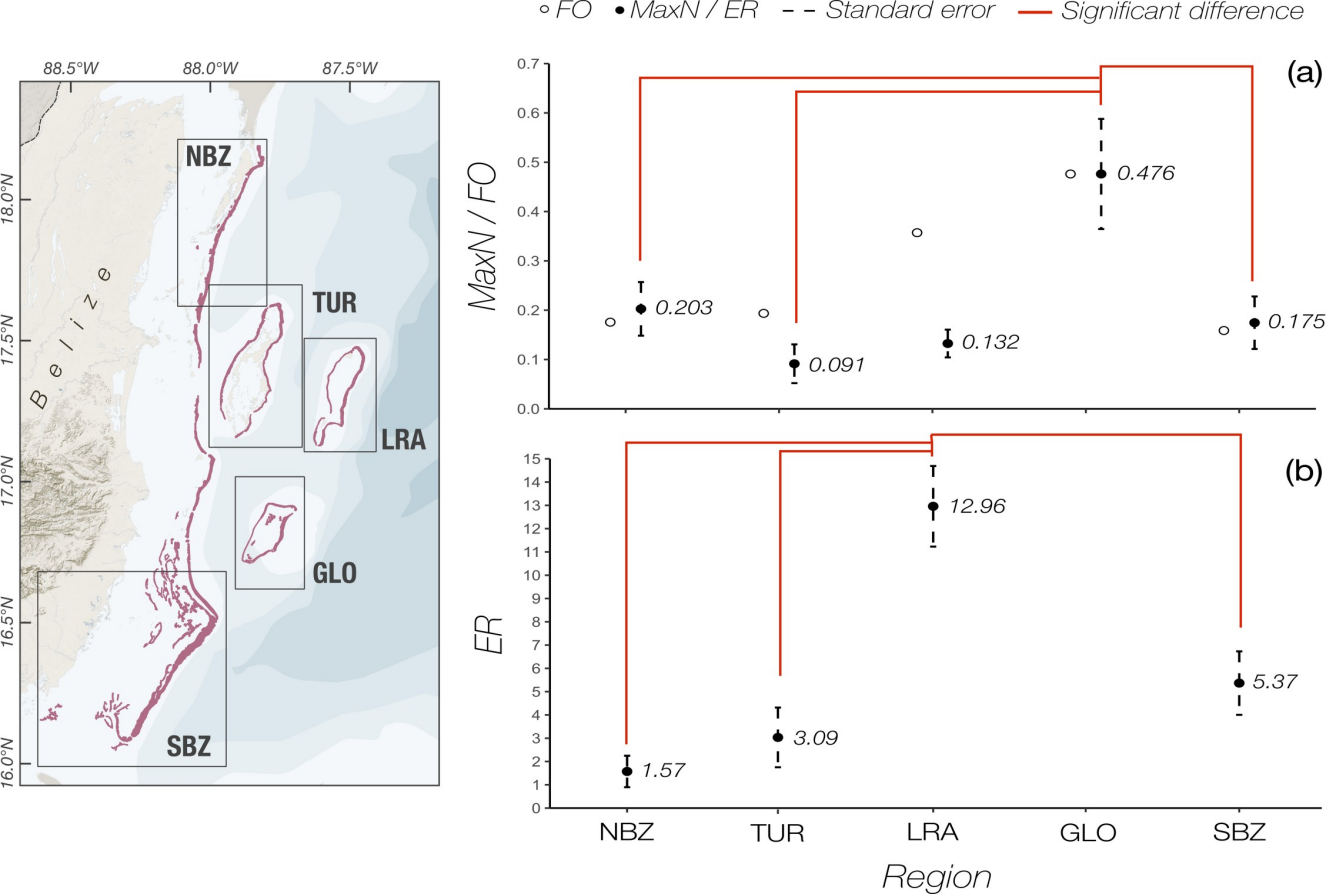
Distribution of abundance of nurse sharks (*Ginglymostoma cirratum*) in Belize. (a) Mean MaxN and Frequency of Occurrence (FO; black and empty dots, respectively), and (b) encounter rate (ER, sharks km^-2^) of nurse sharks in each study region obtained from BRUVs and transects, respectively. Bars represent standard errors. Red lines represent pairs of significantly different estimates. Glover’s reef does not have data in panel (b) as transects were not conducted at that site. GLO = Glover’s reef, LRA = Lighthouse reef, NBZ = North Belize, SBZ = South Belize, TUR = Turneffe Atoll. Map base layer created in MapBox Studio (https://studio.mapbox.com) using freely available data from MapBox (hillshade and terrain data; can be found within the software) and Natural Earth (www.naturalearthdata.com; bathymetry and geopolitical contours).). Barrier reef (pink) shapefile data was obtained from http://www.biodiversity.bz (2015 version; [36]).

**Fig 4:**
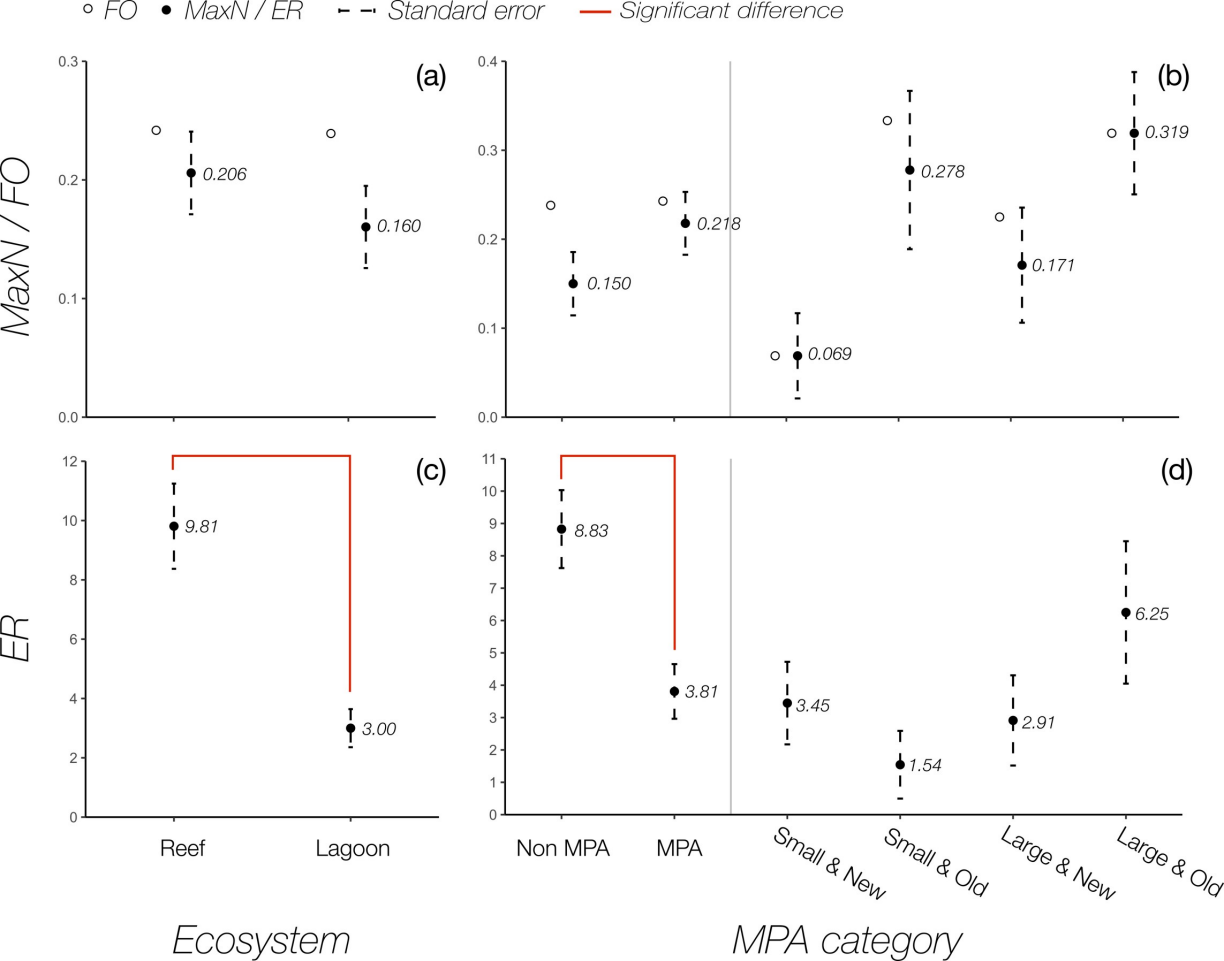
Effect of protected areas and ecosystem type on the abundance of nurse sharks (*Ginglymostoma cirratum*) in Belize. Abundance is represented as mean MaxN (black dots) or frequency of occurrence (FO, empty dots) (a, b) for BRUVs and encounter rate (ER, sharks km^-2^; c, d) for transect surveys within and outside marine protected areas (MPA; a,c), in different categories of protected areas (a, c), and different ecosystem types (b, d). Red lines represent pairs of significantly different estimates.

In total, 79 nurse sharks were recorded in 64 of the BRUV deployments (15.6% of total number of deployments; mean N° of nurse sharks per BRUV = 0.193, range 0 to 4). Most sharks were sighted in LRA (n = 32), 15 were sighted in NBZ, 11 in SBZ, 15 at GLO, and 6 at TUR (Table 1). After correcting for multiple years of surveys, mean MaxN was found to be highest at GLO (0.476±0.112 sharks hour^-1^, mean± standard error), and lowest at TUR (0.091± 0.028 sharks hour-1; Fig 3). Differences between regions were found to be statistically significant (Kruskall-Wallis test, χ^2^_4_ = 12.86, p = 0.01). Mean MaxN at GLO was significantly higher than at all other regions (Dunn test, p<0.05) save for LRA (Dunn test, p = 0.412). Differences among all other regions were not found to be significant (Dunn test, p>0.5). Nurse sharks were recorded in 24.3% of deployments made within MPAs, and in 23.8% of deployments made outside MPAs. MaxN at sites within MPAs was somewhat higher than sites outside of MPAs (mean 0.218 ± 0.035 sharks hour^-1^ vs 0.150 ± 0.036 sharks hour^-1^, mean± standard error), but the difference was not found to be significant (Kruskall-Wallis test, χ^2^_4_ = 12.86, p = 0.01). Among MPA categories, old MPAs had a somewhat higher MaxN than younger MPAs and sites outside of MPAs (Fig 4), however there was much variation between sites and no statistically significant difference between categories could be detected (Kruskall-Wallis test, χ^2^_4_ = 8.68, p = 0.07).

Finally, mean MaxN of nurse sharks in reef ecosystems was slightly higher than lagoon ecosystems (0.206 ± 0.035 sharks hour^-1^ vs 0.160 ± 0.035 sharks hour^-1^, mean± standard error; Fig 4), though the difference was not statistically significant in this case either (Kruskall-Wallis test, χ^2^_1_ = 0.054, p = 0.82).

AIC scores identified the best model as that containing only MPA category and human impact gravity, as well as the interactions between them, as explanatory variables of the probability of occurrence of nurse sharks. The resulting model, however, only explained 10% of the total variance, suggesting that other key explanatory factors might have been missed. Adding all other factors in the model only decreased the residual variance by 3%. Among MPA categories, only large and old MPAs were found to have a significant positive effect on the probability of encountering nurse sharks, as did the absence of MPA when compared to small and new, small and old, and large and new protected areas.

**Fig 5:**
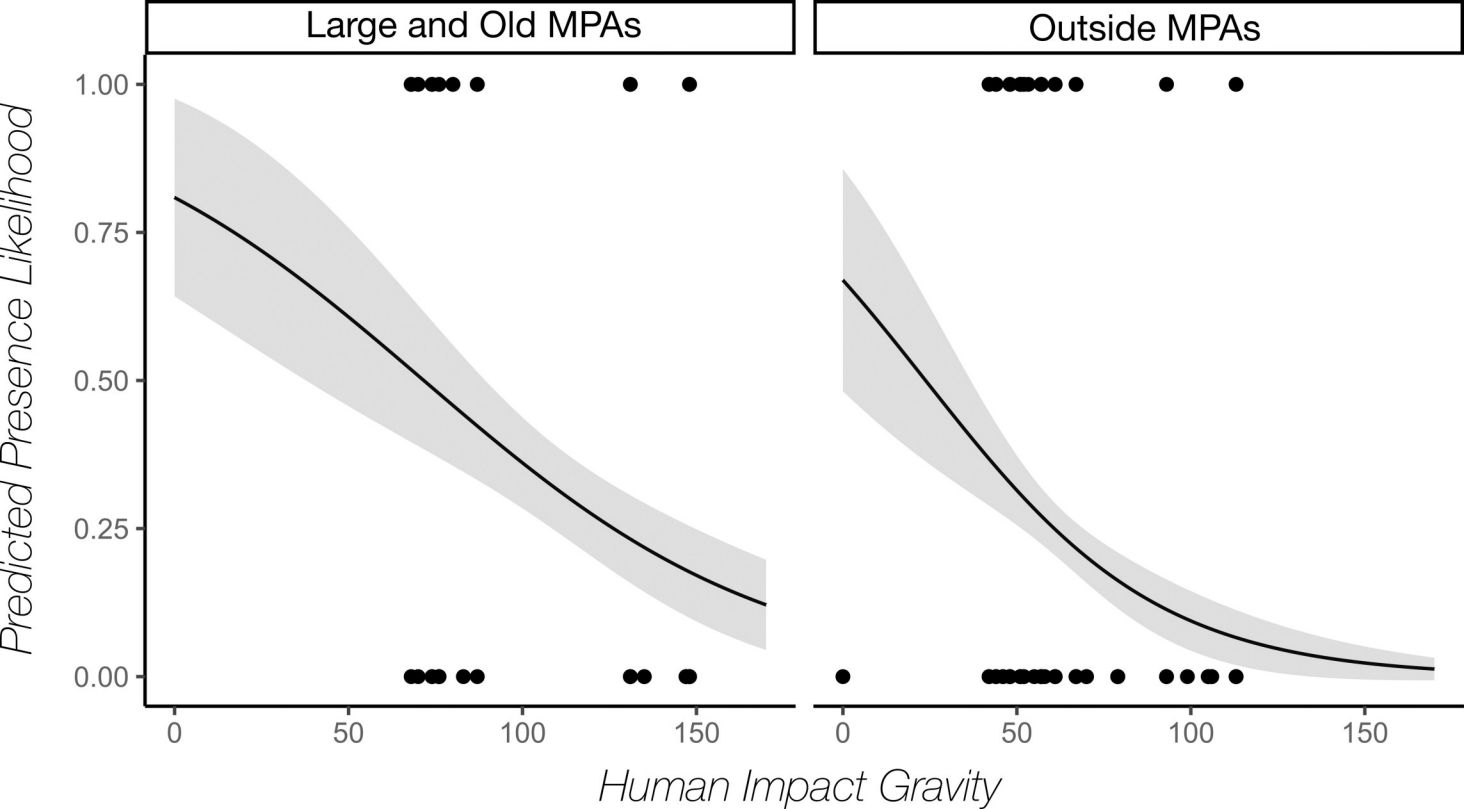
Effect of human impact gravity on likelihood of presence of nurse sharks (*Ginglymostoma cirratum*). Predicted likelihood of nurse shark presence obtained from GLM models as a function of human impact gravity is depicted as solid black line for the two Marine Protected Area (MPA) categories for which the effect of human impact gravity was found to be statistically significant. Grey shading represents confidence intervals given by standard errors. Black dots represent raw presence (1) and absence (0) values on which the model is based for these categories. Large and old protected areas are defined as containing >20km of reef and being >20 years old.

The effect of human impact gravity was only significant outside protected areas and inside large and old MPAs, in which cases it was negatively correlated with the probability of encountering nurse sharks (Fig 5).

### Encounter rate and absolute abundance

Throughout the survey, 211 nurse sharks were recorded in 117 of a total 414 transects, which covered a total area of 24.84 km^2^ (Table 1). Average ER was found to be significantly different between regions (Kruskal-Wallis test, χ^2^_3_ = 55.19, p<0.001). LRA had a significantly higher ER (12.96 sharks per km^2^), than all other region (Dunn test, z = 6.72,4.88,4.91, p<0.01; NBZ, SBZ, and TUR, respectively). The lowest encounter rate was recorded at NBZ (1.57 sharks per km^2^; Fig 3). Transects conducted within reefs resulted in a significantly higher encounter rate than transects conducted in lagoons (Kruskal-Wallis test, χ^2^_1_ = 15.88, p<0.01; Fig 4). Transects in reefs were also found to have a statistically higher mean water depth (9.50 m) than those in lagoons (4.55m; Kruskal-Wallis test, χ^2^_1_ = 85,33, p<0.01).

In contrast to BRUV results, transects outside of MPAs resulted in significantly higher ER than transects within MPAs (Kruskal-Wallis test, χ^2^_1_ = 13.479, p<0–01; Fig 4). Among protected areas, large and old MPAs had a higher mean encounter rate than all other categories (6.25±2.20; Fig 4), though differences between categories were also not statistically significant (Kruskal-Wallis test, χ^2^_4_ = 14.85, p>0.05).

The three key functions selected for the distance sampling analysis estimated similar detection probabilities, which ranged from 0.50 for the hazard rate key function to 0.59 for the uniform key function (Fig 2). As a result, the densities of nurse sharks estimated by the three functions were almost indistinguishable (Table 2). LRA was identified as the area of highest nurse shark abundance, particularly in the reef ecosystem. However, due to a larger area of habitat available, nurse sharks were found to be most abundant at SBZ. TUR and NBZ had lower densities of nurse sharks than the other two regions, in both the lagoon and reef ecosystem.

**Table 2:** Density and abundance of nurse sharks in Belize.

Function	Region	Ecosystem	D	se(D)	N	se(N)	Total N	se(Total N)
UNI	LRA	Reef	30.745	5.980	1476	202	*2684*	*338*
		Lagoon	5.980	1.343	1208	271		
	NBZ	Reef	6.043	3.243	441	237	*594*	*260*
		Lagoon	1.590	1.112	153	107		
	SBZ	Reef	14.389	5.406	3381	1270	*3674*	*1274*
		Lagoon	7.157	2.423	293	99		
	TUR	Reef	8.058	2.968	556	204	*1336*	*581*
		Lagoon	1.831	1.277	780	544		
	Not surveyed	Reef	*14*.*809*	*11*.*496*	*2305*	*1760*	*4150*	*2642*
		Lagoon	*4*.*140*	*4*.*421*	*1845*	*1970*		
HN	LRA	Reef	30.810	4.371	1479	210	*2689*	*346*
		Lagoon	5.993	1.363	1210	275		
	NBZ	Reef	1.594	1.116	442	238	*595*	*261*
		Lagoon	6.056	3.257	153	107		
	SBZ	Reef	14.419	5.443	3388	1279	*3682*	*1283*
		Lagoon	7.171	2.442	294	100		
	TUR	Reef	8.074	2.989	557	206	*1339*	*584*
		Lagoon	1.835	1.282	782	546		
	Not surveyed	Reef	*14*.*840*	*13*.*189*	*2310*	*1773*	*4159*	*2656*
		Lagoon	*4*.*148*	*4*.*032*	*1849*	*1978*		
HR	LRA	Reef	40.193	7.964	1929	382	*2976*	*509*
		Lagoon	5.186	1.666	1047	337		
	NBZ	Reef	7.900	4.387	577	320	*709*	*334*
		Lagoon	1.379	1.015	132	97		
	SBZ	Reef	18.810	7.562	4420	1777	*4674*	*1780*
		Lagoon	6.206	2.539	254	104		
	TUR	Reef	10.533	4.162	727	287	*1403*	*574*
		Lagoon	1.588	1.166	676	497		
	Not surveyed	Reef	*19*.*359*	*15*.*791*	*3013*	*2456*	*4613*	*3081*
		Lagoon	*3*.*590*	*4*.*173*	*1600*	*1860*		

Density (D, sharks per km^2^) and abundance (N) of nurse sharks in each region (Lighthouse Reef, LRA; North Belize, NBZ; South Belize, SBZ; Turneffe Atoll, TUR) estimated from each key function through distance sampling analysis: Uniform with cosine adjustment (UNI), half normal (HN), and Hazard-rate with ecosystem-specific corrections (HR). Density for the region not surveyed was calculated as the mean of densities from surveyed regions. Italics formatting denotes numbers that were not estimated directly from distance sampling analysis.

### Population size estimation

The average encounter rate per unit area of nurse sharks in Belize was 2.13 sharks per km^2^, which translated to a minimum population size estimate of 3,858 nurse sharks. The hazard rate key function used in distance sampling analysis returned the highest estimates of abundance (Table 2), and was therefore used to obtain the maximum likely estimate of population size. Using the values returned by the function, the density of nurse sharks for the areas not surveyed were calculated as 19.36 sharks per km^2^ for reefs and 3.59 sharks per km^2^ for lagoons. The maximum population size was consequently estimated to be 14,375 sharks.

## Discussion

This study represents the first attempt to characterise the abundance and distribution of nurse sharks at a countrywide scale. Five regions of the Belizean reef and coastal area, including all three offshore atolls and the barrier reef divided into two sections, were surveyed for nurse sharks from 2012 to 2017. Nurse sharks were observed in every region of the Belizean reef that was surveyed, indicating that this species is likely ubiquitous in the country. Nonetheless, important differences in relative abundance between regions could be identified, especially between the more remote atolls (LRA and GLO) and the barrier reefs.

GLO was characterised by a significantly higher MaxN and FO than all other regions. The abundance of nurse sharks at GLO had previously been highlighted by a fisheries-independent survey, which found nurse sharks to constitute 68% of the elasmobranch catches made from longlines here [18]. Another study also reported GLO as the area of highest relative abundance (measured as number of sharks per hour from BRUV deployments) in Belize [48]. GLO is an internationally recognised key area for marine conservation (Designated as a UNESCO world heritage site in 1996), hosting some of the reefs of highest diversity in the Caribbean [49,50]. This site was also declared a marine reserve in 1993 and is the largest MPA in the country. Altogether, the good health of the atoll and its protected status can explain the high density of nurse sharks observed here.

TUR was instead highlighted to be an area of low nurse shark density by both BRUVs and transect surveys. Although TUR is also a protected area, its establishment as a marine reserve only occurred in 2012 with management and enforcement starting in April 2014. The broad use of nets in the lagoon and reef passes that were observed to capture nurse sharks (R Graham pers obs), and the high number of fishers along with the Atoll’s proximity to Belize City, suggests intense exploitation of nurse sharks in this region before the introduction of the national ban and the implementation of the marine reserve’s management. Given the low abundance of nurse sharks recorded, and their slow life history [51], it is possible that the atoll’s population has not yet recovered. Comparisons of mean MaxN at Turneffe Atoll after 2, 3, and 4 years since its declaration as a marine reserve showed no signs of an increase in abundance of nurse sharks in the Atoll.

LRA, the easternmost atoll in Belize, was found to have intermediate mean MaxN between GLO and TUR, similarly to NBZ and SBZ. However, the ER and FO at LRA were found to be the highest and second highest, respectively, of all three other regions surveyed by transects (TUR, NBZ, and SBZ). LRA has a similar habitat composition to GLO (lagoon to reef area ratio = 0.214 and 0.225, respectively), and is similarly isolated from the mainland. In contrast, NBZ and SBZ are closer to human settlements and might have suffered higher impacts from anthropogenic activities. LRA also had the lowest mean human impact gravity of all regions. It is thus likely that the higher ER at LRA is a better indicator of local relative abundance than MaxN. MaxN at sites along the barrier reef of Belize was also found to be intermediate between LRA and GLO in a study by Clementi and colleagues [48], although the areas surveyed by the study differed from those explored here.

Overall, differences in abundance of nurse sharks seemed to be mainly attributable to differences in protection level and human impact. Indeed, MPA category and human impact gravity were found to be the strongest predictors of presence of nurse shark in BRUVs. Notably, only large and old protected areas were found to have a significant positive effect on nurse shark presence, while higher human impact gravity scores were associated with lower probabilities of nurse shark presence. These findings are reflected in the high MaxN and ER measured at GLIO and LRA, and the intermediate abundance scores of SBZ, which had the highest human impact than all regions but also a large and old MPA covering its reefs.

Although no overall difference in MaxN between sites within and outside of MPAs could be detected, and ER was even found to be higher outside of MPAs than inside, the overall importance of sustained protection may have been confounded by the amalgamation of many protected areas in the analysis. Large and old MPAs stood out as having a fairly higher nurse shark abundance than all other MPA categories, and similar or higher abundance than non-protected sites. These results are in apparent concordance with the findings of Bond and colleagues [52], who found Caribbean reef sharks (*Carcharhinus perezi*) to be more abundant in protected areas, and of Clementi [48], who also recorded a significantly higher CPUE of nurse sharks within protected areas than at fished sites in Belize. Bothe these studies focused on fairly large and old MPAs, and in concomitance with study further demonstrate the importance of area and time of enforcement for the positive effects of spatial protection to emerge [34,35].

While a general agreement in patterns of relative abundance was found between BRUV and transect data, these methodologies also produced some considerably different estimations for certain regions, ecosystems, or protected areas types. Most notably, transects found nurse sharks to be significantly and conspicuously more abundant in reef systems, while no differences could be detected in MaxN among BRUVs deployed in reefs and lagoons. These differences likely arise from a combination of confounding factors, including the nurse shark’s biology, their responses to human disturbance, and observer’s error. Nurse sharks are generally inactive during daylight hours (when surveys were conducted; [53]), which could bias BRUV sightings if it the use of bait was not sufficient to incentivize sharks to move, especially in reef systems where they preferentially rest. Conversely, this could have made nurse sharks less likely to be spotted in transects within the lagoon, where the species is mor likely to be moving. At the same time, survey sites in the lagoon ecosystem were found to be significantly shallower than those in reef ecosystems. It is hence possible that transect surveys in shallower waters could have led to nurse sharks more likely feeing the approach of observers in lagoons, in turn inflating the difference between ecosystems in transect surveys. Finally, seafloor complexity and water visibility could have affected the observer’s ability to detect sharks in transects and BRUV footage.

It wasn’t possible in this study to identify a clear relationship between MaxN or ER and depth, or to quantify the effect of substratum complexity on sighting probability, and it is therefore not possible to clearly state which method produced more accurate estimations of abundance. This study therefore highlights the importance of combining different techniques to obtain a more complete and accurate description of a species’ distribution and relative abundance.

The total population size of nurse sharks for Belize was estimated to be at least 3,858 individuals, and could be as high as 14,375 individuals. Although it is unlikely that the real nurse shark population size falls under the conservative estimate of 3,858 individuals, less certainty is associated with the upper limit of the range. The area of suitable nurse shark habitat used here to infer population size is also likely to be an underestimation; the total population of nurse sharks is therefore likely to be closer to the upper limit proposed here. There are no other national estimates of nurse shark abundance, but an estimate for Atol das Rocas, Brazil (the only atoll found in the Southern Atlantic Ocean) by Castro and Rosa (2005, [16]) using mark-recapture techniques estimated the population size of nurse sharks to be 369 individuals, which translates to a density of almost 40 sharks per km^2^ for the atoll. The reef ecosystem in LRA was found to have a similar density (40.2 sharks km^-2^), while all other regions surveyed in the present study were found to have a much lower absolute density than Atoll das Rocas. While these offshore reefs have not been completely spared from human exploitation, it is possible that the population density measured here is close to the natural state, and might represent a target density for conservation measures aimed at re-establishing nurse shark populations. Replication of similar studies in other isolated systems, as well as close monitoring of nurse shark populations rebound in other Belizean Atolls will provide additional parameters against which to test this hypothesis.

Future transect surveys conducted in the sections of the Belizean reef that could not be covered in this study (i.e. the central barrier reef and GLO) should help to refine the figures presented here. For example, an extremely low density of nurse sharks in NBZ was reported and this could be a result of the fact that the surveys did not specifically cover the three main feeding aggregations of nurse sharks present in the region. The degree of residency of the sharks frequenting the aggregations is not known, and is therefore difficult to determine whether these individuals could have been encountered in surveys conducted nearby the aggregations. The number of sharks visiting the central provisioned aggregation is estimated to be in the range 100–150 (MarAlliance, unpublished data); thus, although the addition of these sharks to the population estimate presented here for NBZ would significantly increase nurse shark count for the region, it would not change considerably the population estimates presented for the entire country.

It is important to note that the figures reported in this study rely on the crucial assumptions that nurse sharks are mostly resident in the country and do not regularly move between regions. While there is evidence to suggest that nurse sharks in Belize are residential and only seldom cover distances of 10km or more [18], other studies conducted in the region have revealed that the species is capable of long-distance migrations [29,30]. Further characterisation of the movements of these sharks in Belize is needed to ascertain that the population fulfils the residency requirements of this study. Capture-Mark-resighting studies could provide such information. This methodology has been previously applied to nurse sharks and other elasmobranch before, with successful results [16,54–56]. Thanks to the highly developed diving industry of Belize [57] there is high potential for the development of a citizen-science programme that will allow for the collection of high amounts of photographic records that can be used for this purpose. More refined high-resolution data could also be obtained through a variety of telemetry methods, including both passive acoustic tracking and high-resolution satellite tracking.

In light of the ongoing threats (both direct and indirect) that nurse sharks face in Belize, it is important to obtain information on the status and trend of the national population size that can inform future conservation and development planning. This study offers a baseline for the long-term monitoring of the Belizean nurse shark population and suggests areas of importance for future research. Particular attention should be given to the two atolls furthest from the mainland (LRA and GLO), as they appear to host the highest abundance of nurse sharks in the country, as well as having experienced lower levels of anthropogenic disturbance, probably due to their isolated location. Since surveys were initiated shortly after the introduction of a country-wide ban on nurse shark fishing, this data also offers a useful comparison for future studies aimed at quantifying the success of the national ban. Given the paucity of data available for this species, the data presented here improves our understanding of nurse sharks in the wider Caribbean basin, providing a measure of comparison for future studies in other regions. Further standardized population assessments in other countries and regions of the Western Atlantic are strongly recommended in order to obtain a complete picture of the conservation status of nurse sharks in the region.

## Supporting information

S1 Table Selection scores of models explaining nurse shark (*Ginglymostoma cirratum*) likelihood of presence from BRUV deployment data. (DOCX)

S1 File Effort and sightings of nurse sharks in Belize. The excel file contains 4 sheets, detailing the BRUV efforts (sheet 1) and sightings (2) and the transect effort (3) and sightings (4).
(XLSX)
