# Supplementary material for: Nation-wide assessment of the distribution and population size of the data-deficient nurse shark (Ginglymostoma cirratum)
Source: PLoS One. 2021 Aug 24;16(8):e0256532. doi: 10.1371/journal.pone.0256532 (PMC8384212; doi:10.1371/journal.pone.0256532)
Supplement: S1 Table — (DOCX) [file pone.0256532.s001.docx]

| **Model specification** | **AIC** | **𝚫AIC** | **Deviance** |
| --- | --- | --- | --- |
| PL ~ MPA Category * Human Gravity Impact + Depth + Location + Ecosystem | 275.30 | / | 243.30 |
| PL ~ MPA Category + Human Gravity Impact + Depth + Ecosystem type+ MPA Category:Human Gravity Impact | 270.58 | 4.72 | 246.58 |
| PL ~ MPA Category + Human Gravity Impact + Depth + MPA Category:Human Gravity Impact | 268.75 | 1.83 | 246.75 |
| **PL ~ MPA Category + Human Gravity Impact + MPA Category:Human Gravity Impact** | 267.05 | 1.7 | 247.05 |

**Table S1 – Selection scores of models explaining nurse shark (*Ginglymostoma cirratum*) likelihood of presence from BRUV deployment data.**

PL= Presence likelihood. Final selected model is highlighted in bold.
